# Acute idiopathic blind spot enlargement syndrome in a patient with high myopia: A case report with multimodal imaging findings

**DOI:** 10.1097/MD.0000000000051426

**Published:** 2026-10-30

**Authors:** Hanzhi Liu, Zhengfeng Liu, Qiuxin Wu, Yifei Yang, Xuemei Pan

**Affiliations:** a Ophthalmology & Optometry Medical School, Shandong University of Traditional Chinese Medicine, Jinan, Shandong Province, China; b Shandong University of Traditional Chinese Medicine, Jinan, Shandong Province, China; c Affiliated Eye Hospital of Shandong University of Traditional Chinese Medicine, Shandong Academy of Eye Disease Prevention and Therapy, and Shandong Provincial Key Laboratory of Integrated Traditional Chinese and Western Medicine for Prevention and Therapy of Ocular Diseases, Jinan, Shandong Province, China.

**Keywords:** acute idiopathic blind spot enlargement syndrome, high myopia, optical coherence tomography, outer retinopathy, visual field defect

## Abstract

**Rationale:**

To report the clinical manifestations, multimodal imaging features, and treatment outcomes of a case of acute idiopathic blind spot enlargement syndrome (AIBSES) in a patient with high myopia.

**Patient concerns:**

A 47-year-old male with high myopia presented with sudden visual field (VF) obscuration and photopsia in the left eye, with best-corrected visual acuity of 0.05 in the affected eye at initial examination.

**Diagnoses:**

Based on characteristic VF findings showing diffuse central sensitivity depression with a temporal defect contiguous with the physiological blind spot, optical coherence tomography (OCT) showing heterogeneous reflectivity of the neurosensory retina and focal discontinuity of the ellipsoid zone (EZ) signal, and multifocal electroretinography demonstrating reduced P1 response amplitude density across all 5 tested rings, a diagnosis of AIBSES was established.

**Interventions:**

The patient received systemic corticosteroid therapy, followed by additional pharmacologic treatment and adjunctive acupuncture at the 4-month follow-up.

**Outcomes:**

At the 7‑month follow‑up, best-corrected visual acuity improved from 0.05 to 0.2, with substantial improvement in the VF defect and gradual restoration of EZ continuity on OCT. Because spontaneous recovery is recognized in AIBSES, the observed improvement cannot be attributed solely to corticosteroid therapy.

**Lessons:**

AIBSES is an outer retinopathy characterized by acute VF defects, photopsia, and outer retinal structural abnormalities, with visual dysfunction that may be disproportionate to relatively subtle fundoscopic findings. Characteristic VF defects and EZ disruption on OCT are important diagnostic features. AIBSES should be considered in patients presenting with acute visual decline or VF defects but minimal fundus abnormalities to avoid misdiagnosis as optic neuritis or other disorders. In patients with high myopia, myopia-related structural and functional alterations may influence the interpretation of ancillary examinations; therefore, comprehensive evaluation based on multimodal imaging and electrophysiological assessments is essential.

## 1. Introduction

Acute idiopathic blind spot enlargement syndrome (AIBSES) was first described by Fletcher et al in 1988 as an acute ocular disorder primarily involving the peripapillary outer retina.^[1]^ It is typically unilateral and is considered a phenotypic variant within the spectrum of acute zonal occult outer retinopathy (AZOOR).^[2,3]^ Although its etiology remains unclear, the predominance of AIBSES in young women suggests potential involvement of inflammatory and autoimmune mechanisms.^[4]^

The diagnosis of AIBSES relies on characteristic clinical presentation and multimodal assessment, including visual field (VF) testing, fundus autofluorescence, optical coherence tomography (OCT), and electrophysiological examinations.^[4–6]^ Among these ancillary tests, OCT is regarded as the most critical imaging modality for diagnosis, as it can directly visualize the disruption of outer retinal structures, particularly the ellipsoid zone (EZ) surrounding the optic disc. Due to a lack of clinical awareness, AIBSES is frequently misdiagnosed as optic neuritis. Currently, no standardized treatment protocol exists; while some cases are self-limiting, others may result in permanent VF defects.

Patients with high myopia often exhibit structural alterations of the retina and changes in visual function, which may complicate the interpretation of ancillary examinations. However, reports describing the clinical manifestations and multimodal imaging characteristics of AIBSES in the setting of high myopia remain limited. Herein, we report a case of unilateral AIBSES in a patient with high myopia, aiming to improve clinical recognition of this rare disorder and provide insights for the diagnosis and differential diagnosis of similar cases.

## 2. Case presentation

A 47-year-old male with a history of high myopia presented in July, 2025 with a 2-day history of VF defect and photopsia in the left eye. His medical history was unremarkable, with no prior ocular surgery, recent infection, or autoimmune disease.

On initial examination, the uncorrected distance visual acuity was 0.03 in the left eye, with a best-corrected visual acuity (BCVA) of 0.05 (−10.50/−1.25 × 91°). Intraocular pressure and anterior segment findings were normal. Pupillary examination demonstrated findings consistent with a left relative afferent pupillary defect (RAPD).

Funduscopy of the left eye revealed well-defined optic disc margins, mild optic disc pallor, and no obvious optic disc edema. A tessellated fundus appearance consistent with high myopia was also observed (Fig. 1A). Fundus autofluorescence showed peripapillary hyperautofluorescence in the left eye (Fig. 1B). VF testing demonstrated diffuse central sensitivity depression, accompanied by a temporal VF defect contiguous with the physiological blind spot (Fig. 2A). OCT of the left eye showed an irregular posterior contour of the ocular wall and thinning of the superior and inferior retinal nerve fiber layer, which were considered to be associated with high myopia. Meanwhile, the foveal contour was preserved, with heterogeneous reflectivity of the neurosensory retina and focal discontinuity of the EZ signal (Fig. 3A). Multifocal electroretinography (mf-ERG) revealed a mild reduction in response amplitude density in the central ring (ring 1), severe reductions in rings 2 and 3, a moderate reduction in ring 4, and a severe reduction in ring 5 (Fig. 4). Pattern visual evoked potentials (P-VEP) showed abnormalities that varied according to check size. Under 1.0° check size stimulation, P100 latency was prolonged in both eyes, whereas reduced amplitude was observed only in the left eye. Under 15 arcmin (0.25°) check size stimulation, N75–P100 amplitudes were reduced in both eyes; P100 latency was prolonged in the left eye, whereas the right eye showed intertrial variability, with one prolonged and one shortened measurement (Fig. 5). Magnetic resonance imaging (MRI) of the optic nerves revealed no significant abnormalities.

**Figure 1.**
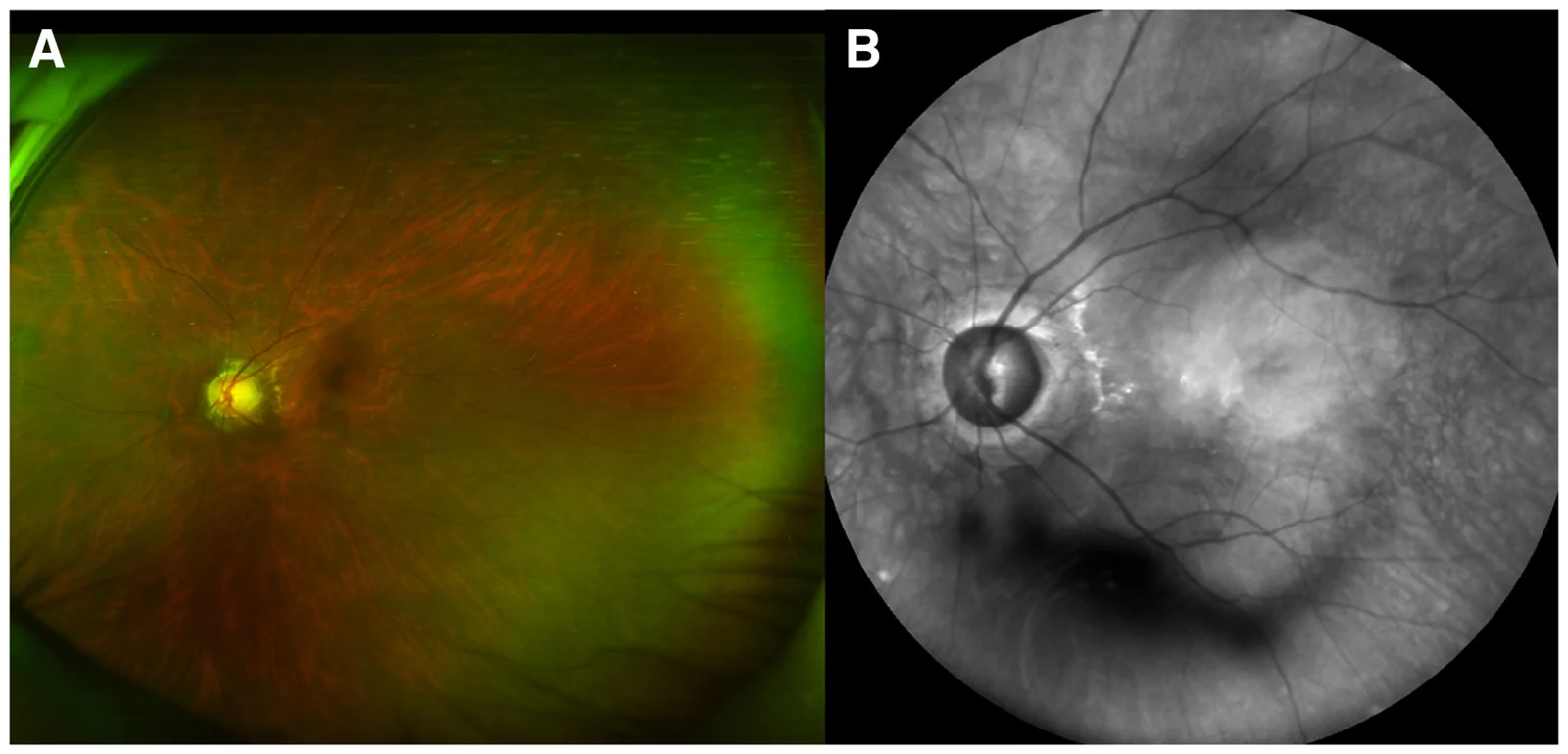
Fundus photography and fundus autofluorescence. (A): The fundus photograph of the left eye shows clear optic disc margins with slightly pale optic disc coloration. (B): The fundus autofluorescence image shows peripapillary hyperautofluorescence in the left eye.

**Figure 2.**
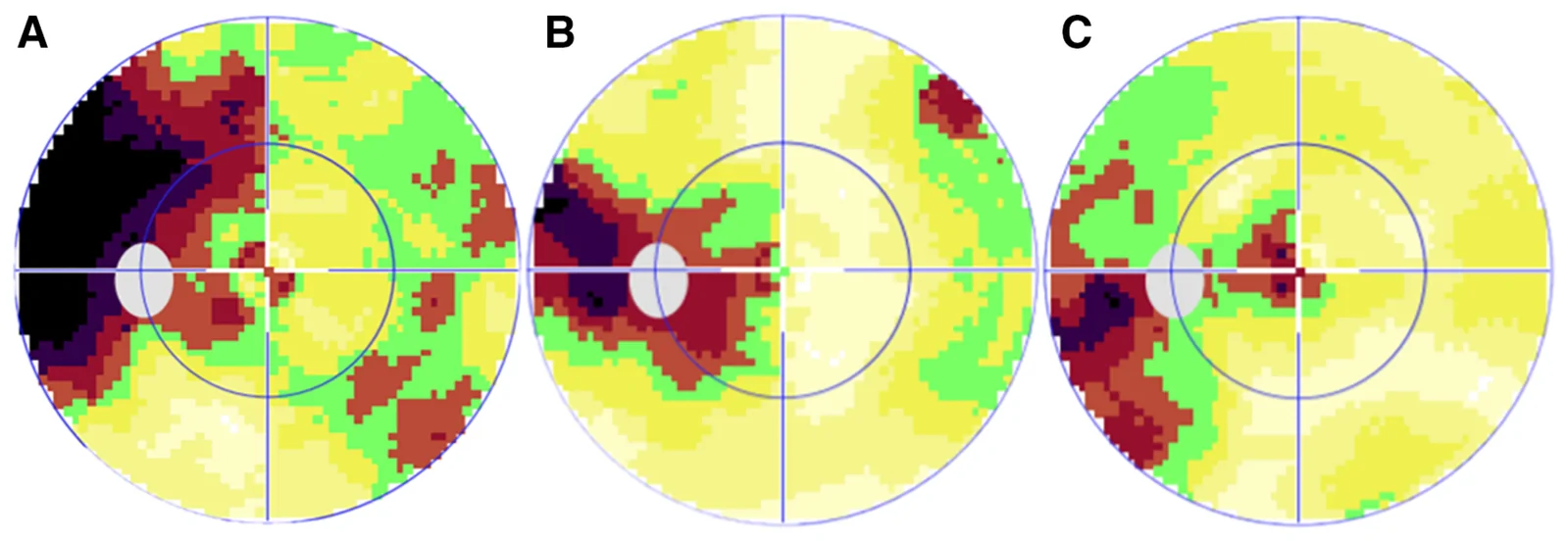
Serial visual field examinations of the left eye. Images were obtained at (A) initial presentation in July 2025, (B) the 4-month follow-up in November 2025, and (C) the 7-month follow-up in February 2026. The visual field defect contiguous with the physiological blind spot gradually improved during follow-up. VF = visual field.

**Figure 3.**
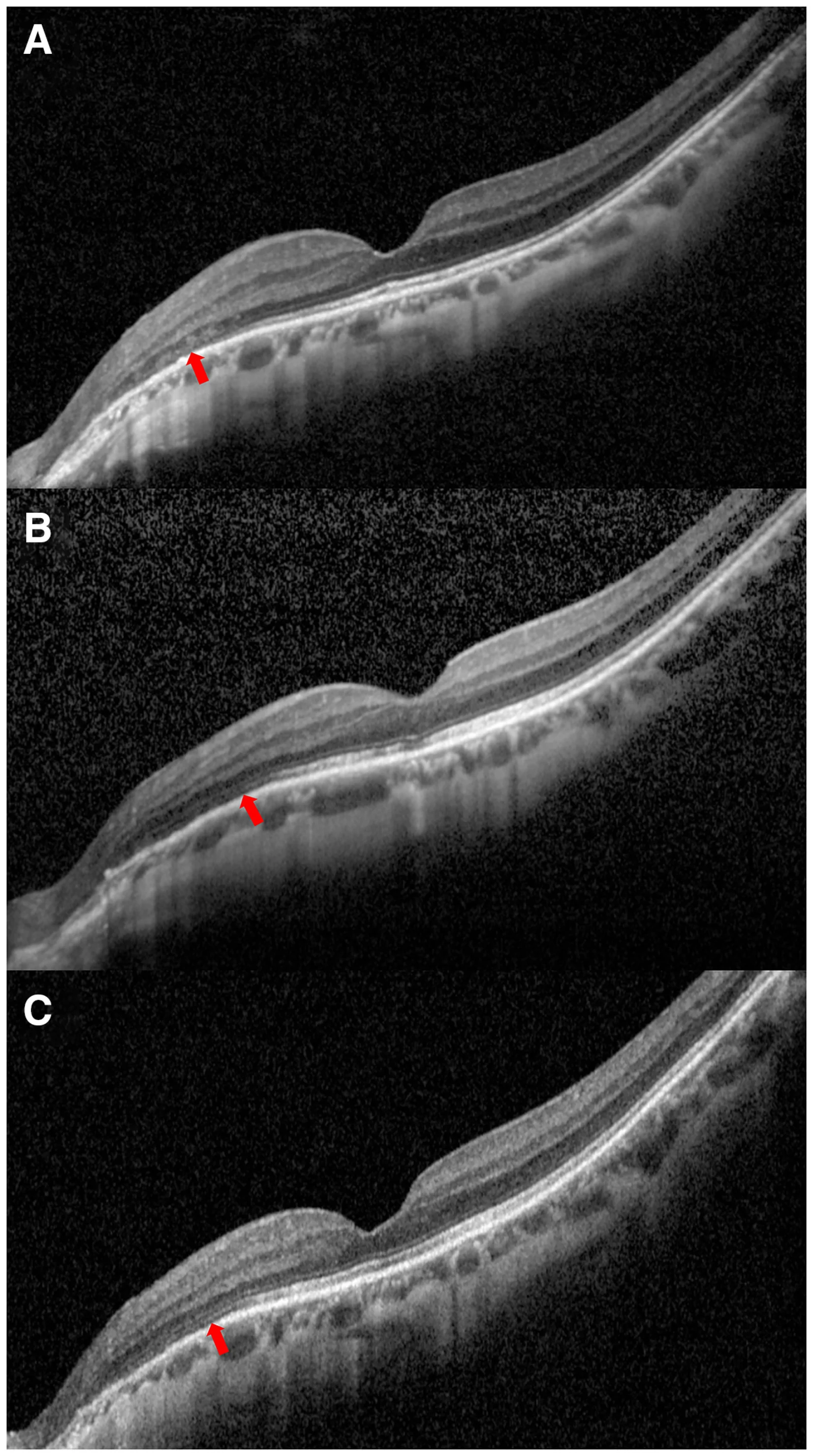
Serial optical coherence tomography findings of the left eye. Images were obtained at (A) initial presentation in July 2025, (B) the 4-month follow-up in November 2025, and (C) the 7-month follow-up in February 2026. The ellipsoid zone (EZ) showed focal disruption at baseline and gradual restoration of continuity during follow-up. OCT = optical coherence tomography.

**Figure 4.**
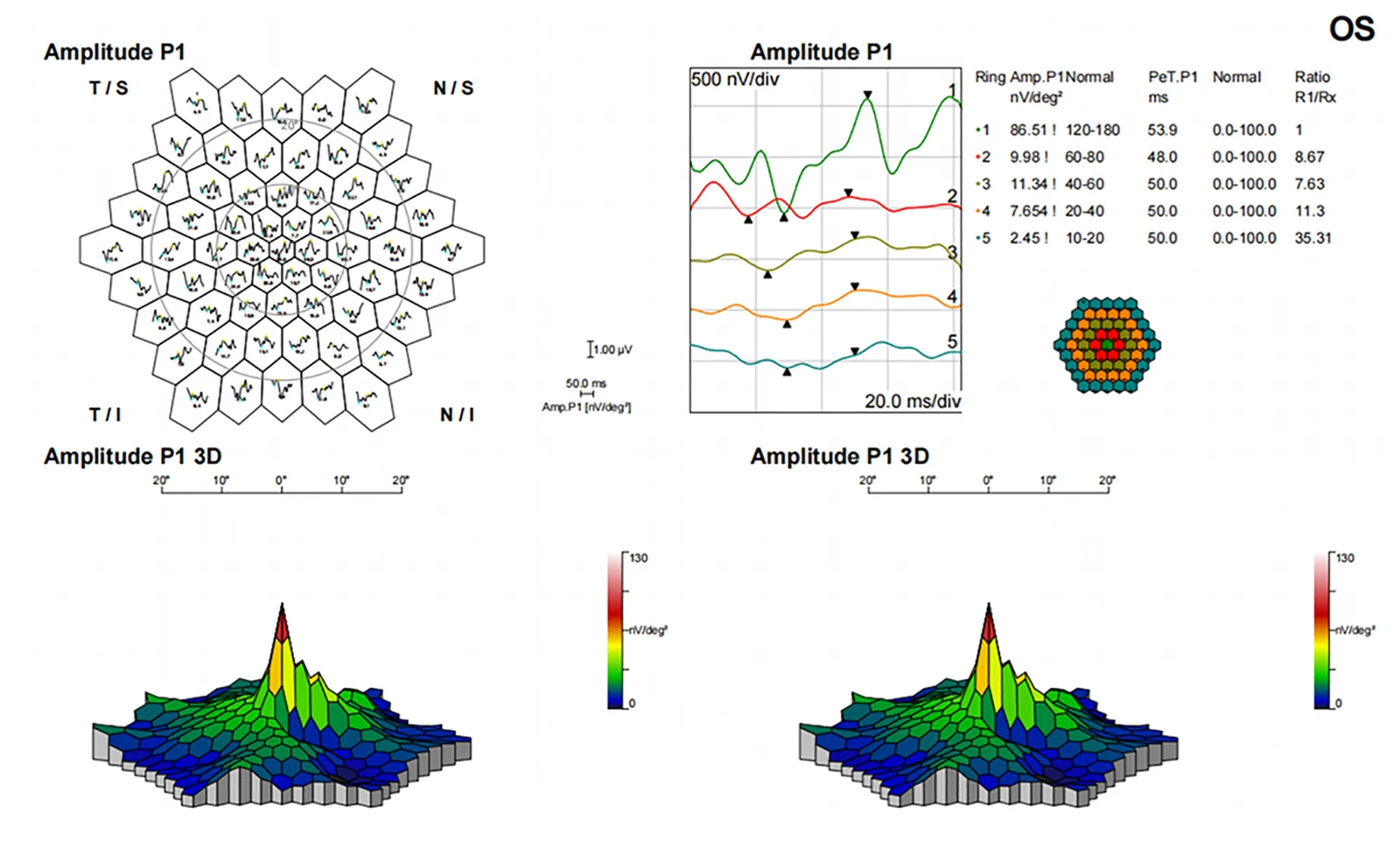
Three-dimensional response density topography of multifocal electroretinography. In the left eye, a mild decrease in ring 1 (central macula), a severe decrease in rings 2, 3, and 5, and a moderate decrease in ring 4 were observed. mf-ERG = multifocal electroretinography.

**Figure 5.**
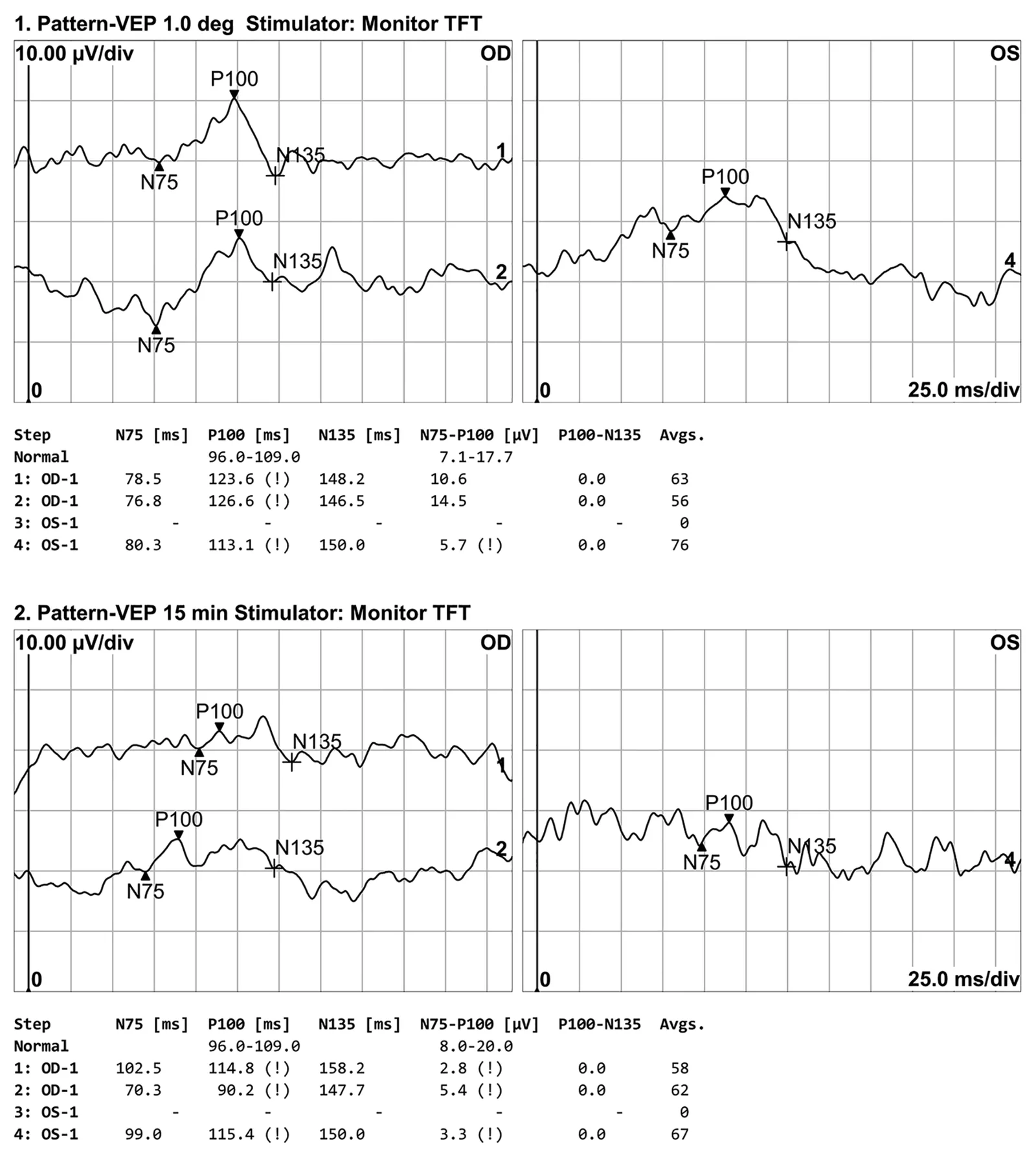
Pattern visual evoked potential (P-VEP) findings. Under 1.0° check size stimulation, P100 latency was prolonged in both eyes, whereas reduced amplitude was observed only in the left eye. Under 15 arcmin (0.25°) check size stimulation, N75–P100 amplitudes were reduced in both eyes; P100 latency was prolonged in the left eye, whereas the right eye showed intertrial variability, with one prolonged and one shortened measurement. P-VEP = pattern visual evoked potential.

Given the acute unilateral visual disturbance and the presence of RAPD, an afferent visual pathway disorder was initially suspected, with left optic neuritis considered in the differential diagnosis. Based on this initial clinical suspicion, the patient received intravenous methylprednisolone sodium succinate at 500 mg/d for 3 consecutive days. However, subsequent multimodal evaluation did not support a primary optic nerve disorder. MRI revealed no significant abnormalities of the optic nerves, whereas VF testing demonstrated a temporal defect contiguous with the physiological blind spot. OCT revealed focal disruption of the EZ, and mf-ERG demonstrated regional retinal dysfunction in the affected eye. Based on these findings, the final diagnosis of AIBSES was established. The patient was discharged on oral prednisone at 50 mg/d, with gradual tapering by 10 mg every 5 days until discontinuation. At the end of this initial course, the left eye remained 0.03, and BCVA improved to 0.1 (–12.00 DS).

At the 4-month follow-up, the patient was readmitted for additional treatment, including circulation-improving agents, neurotrophic medications, and adjunctive acupuncture. The left eye BCVA improved to 0.15 (–12.75/–0.25 × 106°). At 7 months after initial presentation, BCVA further improved to 0.2 (–13.25 DS). Follow-up VF testing showed substantial improvement in the VF defect (Fig. 2B and C), and OCT demonstrated gradual restoration of EZ continuity (Fig. 3B and C). Because spontaneous recovery is recognized in AIBSES and multiple interventions were administered, the independent contribution of each treatment could not be determined.

## 3. Discussion

AIBSES is a rare disorder characterized by damage to the outer retinal structures. The diagnosis of this syndrome relies primarily on characteristic clinical presentation and multimodal imaging findings. The classic clinical features include acute onset photopsia, VF defects, and enlargement of the physiological blind spot.^[1,3,4]^ In the present case, the patient presented with acute VF disturbance and photopsia, and VF testing demonstrated an enlarged blind spot with a contiguous temporal defect, which aligns with the core manifestations of AIBSES. OCT is one of the most important ancillary examinations for establishing the diagnosis of AIBSES, as it can directly visualize abnormalities in the outer retinal structures, particularly disruption or irregularity of the EZ.^[5,6]^ In this case, OCT revealed heterogeneous reflectivity of the neurosensory retina and focal discontinuity of the EZ, findings that are consistent with previously described OCT abnormalities in AIBSES.^[5,6]^ Furthermore, mf-ERG provides an objective assessment of regional retinal function.^[4,6]^ In this patient, mf-ERG demonstrated reduced P1 response amplitude density across all 5 tested rings, providing objective evidence of regional retinal dysfunction and further corroborating the diagnosis of AIBSES.

The etiology and pathogenesis of AIBSES remain incompletely understood. The prevailing hypothesis suggests that the disease primarily affects the peripapillary outer retinal structures, particularly the photoreceptor layer and associated outer retinal components.^[4,6]^ Inflammatory or autoimmune mechanisms have been proposed to contribute to the dysfunction of these structures, leading to photoreceptor dysfunction and consequently resulting in VF loss and photopsia.^[4]^ Disruption of the EZ on OCT may indicate impaired photoreceptor integrity in the outer retina. A previous study reported preservation of the external limiting membrane and EZ, accompanied by absence of the cone outer segment tip line.^[5]^

AIBSES and optic neuritis share overlapping early manifestations, including acute visual decline, VF defects, and RAPD, which can lead to misdiagnosis.^[3,4]^ However, RAPD reflects asymmetric dysfunction of the afferent visual pathway and is not specific to optic neuritis. It has also been reported in patients with AIBSES.^[7]^ Therefore, RAPD alone is insufficient to differentiate AIBSES from optic neuritis. The lesion locations and ancillary examination findings differ substantially between these 2 entities. Optic neuritis primarily affects the optic nerve and is often associated with pain on eye movement, marked color vision impairment, and prolonged P100 latency on P-VEP; in some cases, MRI may show optic nerve enhancement or thickening. In contrast, AIBSES is an outer retinal disorder characterized by relatively subtle fundus findings, while characteristic VF changes – namely blind spot enlargement – together with OCT-documented EZ disruption and mf-ERG evidence of reduced macular and peripheral retinal function, are more valuable for diagnosis.^[3,4,6]^

In the present case, optic neuritis was initially considered because of the acute unilateral visual disturbance and the presence of RAPD, rather than on the basis of RAPD alone, and systemic corticosteroid therapy was initiated. However, further examinations revealed no significant abnormalities of the optic nerves on MRI, whereas OCT demonstrated EZ disruption in the left eye and mf-ERG showed reduced retinal function in the macular and peripheral regions. Together with the characteristic blind spot enlargement and contiguous VF defect, these findings supported the final diagnosis of AIBSES rather than optic neuritis. This case further suggests that AIBSES should be considered in patients with suspected optic neuritis who show no significant optic nerve abnormalities on MRI but present with EZ disruption on OCT and retinal dysfunction on mf-ERG. This diagnostic consideration may facilitate accurate differentiation between AIBSES and optic neuritis.

P-VEP findings in the present case require cautious interpretation. Previous reports have described P100 latency within normal limits in patients with AIBSES.^[4]^ However, P-VEP abnormalities were observed in both eyes in the present case. Under 1.0° check size stimulation, P100 latency was prolonged bilaterally, whereas amplitude reduction was observed only in the affected left eye. Under 15 arcmin (0.25°) check size stimulation, N75–P100 amplitudes were reduced bilaterally; P100 latency was prolonged in the left eye, whereas the right eye showed intertrial variability, with one prolonged and one shortened measurement. Thus, the P-VEP findings in this patient were not entirely typical of AIBSES and were not strictly concordant with the clinically unilateral presentation and unilateral outer retinal abnormalities. Previous studies have suggested that high myopia may be associated with alterations in electrophysiological responses, including reduced VEP amplitudes and prolonged latencies.^[8]^ Therefore, the bilateral P-VEP abnormalities in this patient could not be attributed specifically to either AIBSES or optic neuritis and may have been influenced, at least in part, by high myopia. Accordingly, P-VEP findings should be interpreted in conjunction with clinical manifestations, multimodal imaging, and retinal electrophysiological findings rather than in isolation.

The patient also exhibited several structural changes associated with high myopia, including a tessellated fundus appearance, posterior scleral morphological alterations, and retinal nerve fiber layer thinning. These myopia-related structural and functional changes may affect the interpretation of ancillary examinations and increase the complexity of differentiating AIBSES from optic nerve disorders. However, there is currently insufficient evidence to support a direct association between high myopia and the development of AIBSES. In this case, high myopia was considered more likely to influence the interpretation of structural and functional examinations rather than represent an established risk factor for disease occurrence. Nevertheless, OCT-confirmed EZ disruption and mf-ERG evidence of regional retinal dysfunction supported outer retinal dysfunction, which is consistent with the characteristic findings of AIBSES. Therefore, despite the atypical P-VEP findings, the diagnosis of AIBSES was supported by the concordant findings of the acute clinical presentation, characteristic VF defect, OCT evidence of outer retinal disruption, and mf-ERG evidence of regional retinal dysfunction. In patients with AIBSES and concomitant high myopia, myopia-associated retinal nerve fiber layer changes and electrophysiological abnormalities should be interpreted cautiously because they may be influenced by myopia-related structural or functional alterations.

Regarding treatment and prognosis, AIBSES may follow a self-limiting course; however, the efficacy of corticosteroid therapy remains uncertain.^[4,6]^ Long-term OCT observations have demonstrated gradual restoration of outer retinal structures in some patients without pharmacological intervention.^[9]^ Nevertheless, some individuals may experience persistent VF defects, and evidence regarding optimal treatment strategies remains limited.^[4,6]^ In the present case, systemic corticosteroid therapy was administered. At 7 months after initial presentation, BCVA had improved from 0.05 to 0.2, accompanied by substantial improvement in the VF defect and gradual restoration of EZ continuity on OCT. However, because spontaneous recovery has been reported in AIBSES,^[9]^ the improvement observed in this patient cannot be attributed solely to corticosteroid therapy. The therapeutic contribution of the additional treatments administered at the 4-month follow-up likewise cannot be determined from this single case.

This case report has several limitations. First, this was a single case report, which limits the generalizability of the findings. Although the patient was followed for 7 months, longer-term observation is required to determine the stability of the visual and structural recovery. Second, systematic testing for autoimmune antibodies and viral infection markers was not performed, limiting further exploration of potential underlying etiological mechanisms. Therefore, in future clinical practice, patients with suspected AIBSES should undergo comprehensive evaluation incorporating multimodal imaging, electrophysiological examinations, and necessary etiological investigations, with long-term follow-up to further clarify the natural history and therapeutic outcomes of this disease.

## 4. Conclusion

AIBSES is an idiopathic outer retinopathy characterized by acute VF defects and photopsia, often with visual dysfunction disproportionate to relatively subtle fundus findings. The combination of characteristic VF abnormalities and OCT-detected EZ disruption is essential for diagnosis. For patients presenting with acute VF defects accompanied by photopsia but minimal fundus abnormalities, clinicians should consider AIBSES and perform timely VF testing and OCT to avoid misdiagnosis. In patients with concomitant high myopia, myopia-related structural and functional alterations may complicate the interpretation of ancillary examinations; therefore, diagnosis should be based on comprehensive assessment integrating multimodal imaging and electrophysiological findings. Further multicenter studies are needed to clarify the natural history, prognostic factors, and optimal treatment strategies of AIBSES.

## Author contributions

**Investigation:** Hanzhi Liu, Yifei Yang.**Conceptualization:** Zhengfeng Liu, Xuemei Pan.

**Methodology:** Qiuxin Wu.

**Supervision:** Xuemei Pan.

**Writing – original draft:** Hanzhi Liu.**Writing – review & editing:** Zhengfeng Liu, Xuemei Pan.
